# Assist-As-Needed Exoskeleton for Hand Joint Rehabilitation Based on Muscle Effort Detection

**DOI:** 10.3390/s21134372

**Published:** 2021-06-26

**Authors:** Jenny Carolina Castiblanco, Ivan Fernando Mondragon, Catalina Alvarado-Rojas, Julian D. Colorado

**Affiliations:** 1 School of Engineering, Pontificia Universidad Javeriana Bogota, Cra. 7 No. 40-62, Bogota 110231, Colombia; 2 Department of Industrial Engineering, Pontificia Universidad Javeriana Bogota, Cra. 7 No. 40-62, Bogota 110231, Colombia; imondragon@javeriana.edu.co; 3 Department of Electronics Engineering, Pontificia Universidad Javeriana Bogota, Cra. 7 No. 40-62, Bogota 110231, Colombia; catalina_alvarado@javeriana.edu.co (C.A.-R.); coloradoj@javeriana.edu.co (J.D.C.)

**Keywords:** active control, robotic-assisted systems, EMG control, stroke rehabilitation, hand motion rehabilitation, hand exoskeleton orthosis, assist-as-needed system, feedback-fuzzy

## Abstract

Robotic-assisted systems have gained significant traction in post-stroke therapies to support rehabilitation, since these systems can provide high-intensity and high-frequency treatment while allowing accurate motion-control over the patient’s progress. In this paper, we tackle how to provide active support through a robotic-assisted exoskeleton by developing a novel closed-loop architecture that continually measures electromyographic signals (EMG), in order to adjust the assistance given by the exoskeleton. We used EMG signals acquired from four patients with post-stroke hand impairments for training machine learning models used to characterize muscle effort by classifying three muscular condition levels based on contraction strength, co-activation, and muscular activation measurements. The proposed closed-loop system takes into account the EMG muscle effort to modulate the exoskeleton velocity during the rehabilitation therapy. Experimental results indicate the maximum variation on velocity was 0.7 mm/s, while the proposed control system effectively modulated the movements of the exoskeleton based on the EMG readings, keeping a reference tracking error <5%.

## 1. Introduction

Robotic-assisted systems have gained significant interest in movement rehabilitation in the last decade [1,2,3]. When motor disabilities are generated by a stroke event, robotic-assisted systems can significantly improve the intensity and frequency of the treatment. Moreover, they can speed up rehabilitation progress by increasing accurate motion control by enabling continuous monitoring during the therapies [4,5].

Most robotic systems used in post-stroke rehabilitation can be defined according to the characteristics of the hardware and the training modality. The hardware is focused on the mechanical design, while the training paradigm is focused on the human–robot interaction to overcome the main functional components in the recovery [6]. However, a more effective rehabilitation robotic system is obtained when both the hardware and training paradigms are considered in the design process.

Recent studies about robotic-assisted systems for hand motion rehabilitation are mainly focused on the hardware design [7,8,9,10,11,12], rather than on the human–robot interaction [13,14,15]. As for the hardware system, there are two mechanisms used in rehabilitation: end-point structure and exoskeletons [2,16]. The former has only one point of contact with the hand, usually being easier to control and install. The distal phalanges receive the applied forces by devices to assist or resist the movements. The latter has the robot axes aligned with the anatomical axes of the hand and follows hand biomechanics, which results in higher complexity. Therefore, the exoskeleton is an actuated mechanical system designed to follow the movement of the hand [17].

There are five training paradigm categories [6]: passive, active, corrective, resistive, and active-assistance. Depending on the rehabilitation state of the patient, different modalities can be applied. For instance, in a comparative study conducted in [18], the active-assisted modality showed the most consistent improvement in both body and activity function, due to the assessment of the patient to determine if robotic assistance is needed. However, the motion assistance does not usually change according to the subject requirement once the robotic-assisted system takes control of the motion task. In recent years, a new training modality known as assist-as-needed [13,19,20] has emerged, with the purpose of modulating the assistance provided by the exoeskeleton according to the patient’s muscle effort during the therapy.

**Figure 1:**
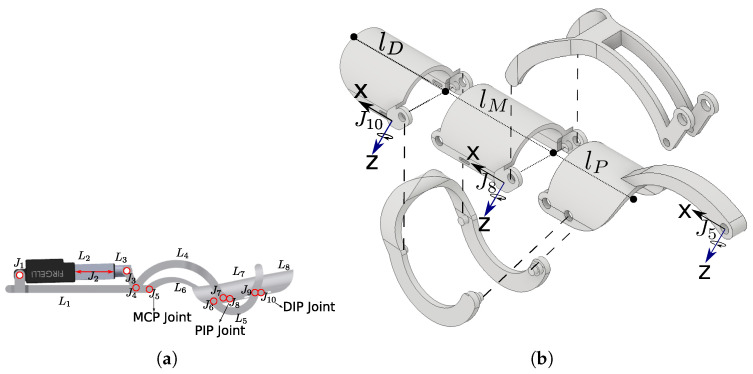
The exoskeleton mechanics for one finger. (**a**) Mechanical structure with a single active DOF, 8-bar, and 10-joint linkage to drive each finger in fully extended and closed configurations. (**b**) Mechanical parts and kinematic frames following the Denavit–Hartenberg (DH) convention.

In this work, a robotic-assisted system is presented where both hardware and training paradigm were considered during the design process. For this purpose, we developed a novel control architecture to provide motion active assistance by following the aforementioned assist-as-needed paradigm. Myoelectric (EMG) and motion signals were used to evaluate the ongoing task performance and the current state of the hand motion, in order to adjust the assistance velocity given by the robotic exoskeleton. The proposed mechanical structure was based on the design originally introduced by Cui et al. in [17], as shown in Figure 1.

In the context of motion rehabilitation, most control methods use EMG signals to either adapt the input references or trigger the assistance. However, following the assist-as-needed paradigm requires the adaptation of the control law. In Teramae et al. [21], EMG signals were used to assist the deficient torque using a model predictive control (MPC) approach, by estimating the patient’s joint torques to generate a target movement that enhances the recovery. Peternel et al. [22] implemented an adaptative feed-forward control where the robot’s joint torque trajectories are adapted according to the patient intention. EMG signals were used to estimate the direction of torque change to minimize muscle effort. Although most of the body of work is oriented at characterizing muscle effort based on measuring muscle force from EMG signals [23,24,25], we found that EMG signals are acquired from agonist and antagonist muscle groups, restricting the degree of freedom of the system for more than two motions. Therefore, EMG-based methods are generally used for low-degree-of freedom exoskeletons.

In previous work [26,27,28], we conducted significant studies to characterize muscle effort by extracting relevant features from the patient’s EMG signals and training machine learning models to identify hand motions according to standard clinical exercises applied for hand rehabilitation. Here, our contribution is twofold: (i) a combined model based on artificial neural networks (ANNs) and fuzzy logic was implemented to determine specific levels of actuation velocities for the exoskeleton, by identifying muscle effort from the EMG signals; (ii) an adaptive control scheme for the exoskeleton with feedback from the ANN-fuzzy model to actively counterbalance muscle effort in real-time.

## 2. Materials and Methods

The proposed robotic-assisted system is mainly composed of the exoskeleton and the assistance controller, according to the assist-as-needed training paradigm.

### 2.1. The Exoskeleton

A hand orthosis was developed based on the mechanical design introduced by Cui et al. [17]. Our system was modified to enable proper assistance for the index, middle, ring, and little fingers. The thumb was not included due to its complex motion, which imposed significant challenges for the mechanical structure.

Each finger of the exoskeleton has a single active degree of freedom (DOF) and three mechanical connections (links) corresponding to the three phalanges. The mechanism has an 8-bar with 10 joints overall, confined to the dorsum of the hand in order to minimize sensory feedback interference and low apparent inertia to the intended subjects. Figure 1a shows the exoskeleton design, where each circle in red represents a rotational joint, while the double-ended arrow represents a prismatic joint. The joints named J10, J8, and J5 correspond to the distal interphalangeal (DIP), proximal interphalangeal (PIP), and metacarpophalangeal (MCP) joints of the human finger. As detailed in Figure 1b, five mechanical pieces compose the finger links, with only three having direct contact with the human phalanges ($l_{p}$, $l_{M}$, $l_{D}$), while the other two are in charge of synchronizing the movement of all the pieces according to the external force received by the linear actuator displayed in Figure 1a.

The entire exoskeletal structure is driven by four linear actuators fabricated by Actuonix. We used the I-series L12 servo linear actuators with embedded encoders to provide position DC voltage value in the range 0–5 V, where 0 V indicates full contraction and 5 V denotes fully extended. The actuator has a stroke length of 30 mm, but we only required 15 mm due to mechanical specifications of the design. It also has a maximum speed of 6.5 mm/s with loads of 40 N. Its light form factor of 34 g makes this actuator suitable for the application at hand. In terms of position control, the resolution of the actuator with respect to changes in the duty cycle is about 2%, i.e., the actuator drives a minimum translation of 0.6 mm.

The human hand can be modeled as a branched-chain of serially coupled rigid bodies, each finger being a serial chain. In this regard, we have applied classical Denavit–Hartenberg (DH) parameters to geometrically model the kinematics of the finger structure, where each link represents the proximal, medial, and distal bones, being the MCP, PIP, and DIP joint frames. We solved the forward kinematics to calculate the fingertip Cartesian motion as a function of the joint’s rotation, while the recursive Newton–Euler formulation was used to solve the inverse dynamics in order to obtain the joint torques. Table 1 presents the modified Denavit–Hartenberg parameters for the finger structures.

### 2.2. Assistance Control

**Figure 2:**
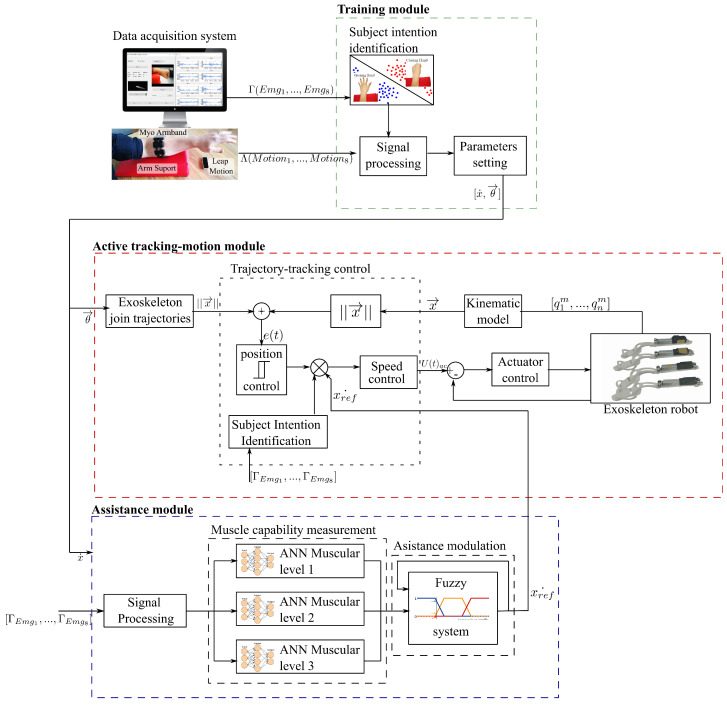
Proposed assistance control architecture based on the “assist-as-needed” paradigm. Three main modules are defined for training, active tracking-motion, and muscle capability measurement.

The assistance control drives the exoskeleton by actively adjusting the output velocity according to the subject’s capability (muscular condition). For this purpose, myoelectric signals are measured to detect any motion and muscle effort changes. The assistance control scheme consists of three modules, as depicted in Figure 2:

1. The training module is an offline process to setup the initial joint positions and velocities of the system. This module enables changing of the parameters used to calculate the trajectory references based on the evolution of the subject throughout the therapy but not during each trial.
2. The active tracking module is a closed-loop position control for the exoskeleton linear actuators. In this regard, the under-actuated joints are driven depending on several factors: the trajectory references, the exoskeleton kinematics, and the assistance commands. This module enables the exoskeleton to track the reference by ensuring smooth and precise positioning.
3. The assistance module is an open-loop assistance control based on an ANN-Fuzzy model that adjusts the output velocity by modulating the reference according to the subject muscular effort detected by the EMG signals. This module enables the dynamic assistance to the subject in order to counterbalance any muscular effort.

A data acquisition system was developed to acquire both myoelectric and motion signals using the Myo Armband and Leap Motion sensors, respectively. Further details are presented in Section 3.

### 2.3. Training Module

This module requires three processes to calculate the initial joint positions and velocities for the system. The first process identifies the subject motion intention from EMG signals to segment the hand kinematic signals in the signal processing step. The identification uses k-NN classifiers with Euclidean distance and 1 neighbor; 10 features in the time domain and 7 in the frequency domain, like mean absolute value, root-mean-square, slope signal changes, waveform length, auto-regressive coefficients, mean frequency, mean power, median frequency, and spectral moments. The z-score standardization method is implemented, and the EMG signals are segmented into windows of 250 ms with overlapping of 60%. The classifiers have an average accuracy of 91% using motion disability information and 93% for healthy motion. Further details can be found in [26].

In the second process the kinematic signals are filtered and interpolated, while the data are segmented and grouped based on the motion position. In the third process, the joint references and initial velocities for the exoskeleton are calculated. The joint angles of the fingers $\vec{\theta }$ are calculated using the direction vectors of the bones according to Equation (1), where $\vec{u}$ is a bone direction vector and *i* is the bone index. The joint angle reference is obtained by calculating the mode across joint angle segments. Finally, the rise time tr is determined from the fingertip Cartesian position to establish the exoskeleton velocity initial value $v_{s}$ using the motor displacement △x, as detailed by Equation (2).

(1) $$\theta_{n}=\cos^{-1}\left(\frac{\vec{u_{(}i-1)}\cdot \vec{u_{i}}}{\left|\right|\vec{u_{i-1}}\left|||\right|\vec{u_{i}}\left|\right|}\right)$$

(2) $$v_{s}=\frac{△x_{act}}{tr_{fingertip}}$$

### 2.4. Active Tracking-Motion Module

This module is a closed-loop that enables the tracking of the joint trajectory by taking into account the velocity reference, the tracking error, and the subject intention. Algorithm 1 describes the entire active tracking control.

Once the reference angles $\vec{\theta }$ are calculated in the former training module, the exoskeleton joint trajectory block calculates the reference trajectories using the kinematic model described in Section 2.1, where a cubic spline interpolation method was used. The end-effector (fingertip) trajectories are obtained for each hand motion through the kinematic model. Then, four operating points are selected to calculate the reference trajectory based on interpolations.

The trajectory tracking block establishes the control signal for the actuator position control according to 4 inputs: the position vector norm ($\left|\right|\vec{x_{ref}}\left|\right|$), the position vector from the kinematics model ($\left|\right|\vec{x_{m}}\left|\right|$), the EMG signal, and the velocity reference ${\dot{x}}_{ref}$ from the assistance module. The control block consists of two main loops working together to establish the actuator position reference. The former loop enables the exoskeleton movement, while the latter sets the actuator reference value. A tracking error e(t) is calculated to track the reference accurately. In this regard, the norm of the position vector of the exoskeleton is calculated and compared against the reference value ($\left|\right|\vec{x_{ref}}\left|\right|$). Then, an on-off scheme receives the error signal and enables the movement under the condition e(t)>threshold. In addition, the actuator control signal $U_{ac}$ is established by using a ramp-signal input, where the slope is the velocity ${\dot{x}}_{ref}$, and the direction is given by the subject intention identification process.

The kinematics model block calculates the position vector of the exoskeleton end-effector based on the actuator displacement. In this regard, a SimMechanics model of the exoskeleton was used to obtain the finger joint angles according to the actuator displacement and the kinematic model. For the exoskeleton mechanics, we used the L12 Actuonix linear motor with an inner position control, allowing for the flexion and extension of the finger. The exoskeleton connections were based on the anatomical attachment of the hand, enabling natural motions and positions, as presented in the previous section.
**Algorithm 1** Active tracking control**Require:  **$\Gamma_{EMG1,...,EMG8}$: EMG vector; $\dot{x_{ref}}$: velocity reference; $\vec{\theta }$: joint angles. 1:  Calculate $\left|\right|\vec{x_{m}}\left|\right|$ and $\left|\right|\vec{x_{ref}}\left|\right|$. 2:  Calculate e(t) = $\left|\right|\vec{x_{ref}}\left|\right|$−$\left|\right|\vec{x_{m}}\left|\right|$. 3:  **if **
e(t)≥1
** then** 4:  $\;U{\left(t\right)}_{ac}={\dot{x}}_{ref}\times sng$; go to 1. 5:  **else**
 6:  
$\;U{\left(t\right)}_{ac}=0$ 7:  **end if** 8:  **if** Motion direction change **then** 9:   Update $\vec{x_{ref}}$ and sng. If the intention is motion 1, sng=1, otherwise, sng=−1.  10:  **end if**  11:  go to 1.


### 2.5. Assistance Module

This module consists of three main blocks: (i) signal processing, (ii) muscle capability measurement, and (iii) assistance modulation.

The signal processing block is in charge of filtering the EMG signals by applying a z-score method and extracting relevant features within 1 s window-time with an overlapping of 30%. The output of the signal processing block is a Γ vector with the elements extracted from the EMG signals. 11 features in the time domain and 4 in the frequency domain were extracted, described as follows: the mean absolute value, root-mean-square, the average of the cumulative trapezoidal numerical integration, zero crossing, the average of spectral density, and 6 multiple time window features (using Hamming, trapezoidal, and Slepian windows), the muscular contraction force, the mean frequency, median frequency, and the power rate. Overall, the feature vector has 148 elements formed by 15 extracted features for the 8 channels of the Myo device sensor, with another 28 features for muscle co-activation. Dimensionality reduction was applied by following the correlation-based feature method.

The second block calculates the degree of membership of the Γ vector to the muscular effort level. It has three feed-forward back-propagation artificial neural network (ANN) models to establish the degrees of membership levels from “not a member” through to “a full member”. The ANN classifiers were trained using the scaled conjugate gradient algorithm due to its efficiency, low memory requirements, and supervised learning. In addition, the number of samples between classes is balanced: 50% corresponds to the main level, and the remaining 50% is divided into the other two levels (25% in each one). Finally, the ANN classifiers have one node in the hidden layer and two classes to predict (belonging or not belonging to the level).

**Figure 3:**
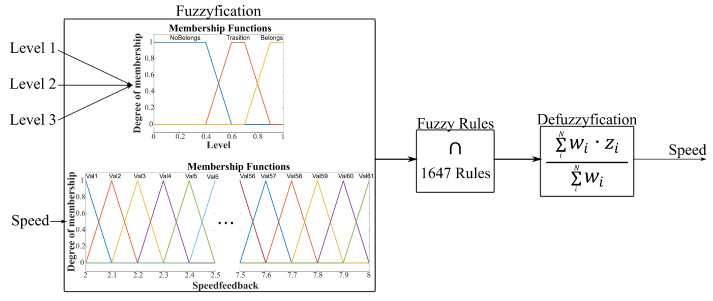
Assistance modulation based on a Sugeno-fuzzy MISO model. Three inputs are from the ANN classifiers corresponding to the muscular level condition, and the other input is the velocity feedback. The output is the velocity reference of the active tracking-motion module.

The assistance modulation block calculates the velocity reference based on the ANN classifier’s outputs and the previous velocity, as presented in Figure 3. A MISO (multiple-input single-output) fuzzy system with velocity feedback was designed and implemented to calculate the output velocity. A logical system is created to describe and model the system’s required behavior. As the first step, the input ranges are divided to establish the necessary combinations. Then, the action rules are designed to train the fuzzy system.

The ANN classifiers have a range from 0 to 1, divided into three states: (i) an input value between 0 and 0.4 means the EMG signal does not belong to the corresponding muscular condition level ($N_{lvl}$), (ii) input value between 0.4 and 0.8 indicates a transition between muscular levels ($T_{lvl}$), and (iii) input between 0.8 and 1.0 means the EMG signal belongs to the corresponding muscular condition level ($B_{lvl}$). As a result, 27 ANN input combinations are obtained (Table 2). In the case of the velocity, it has a range (minimum ${\dot{x}}_{min}$ and maximum ${\dot{x}}_{max}$) defined by the actuator limitations, which is divided into 7 operating points. There are three action rules of the logical system: maintain speed ($S_{p}$), increase speed ($S_{p}+\Delta_{S}$), and decrease speed ($S_{p}-\Delta_{S}$). Table A1 (Appendix A) shows the decisions for the combinations in Table 2 according to the velocity input.

Depending on the combination of the ANN inputs and the speed operating point, the logic system increases, decreases, or maintains the output value. When the ANN inputs indicate that the muscular effort is high and the operating point is low (between 1 and 4), the system will increase the output speed until its stable value while the ANN inputs do not change. Otherwise, the output speed decreases when the ANN inputs indicate minimal muscular effort and the speed is high. For each combination in Table 2 there is a different stable value of output speed. For example, combination s has a stable value at operating point 7, while combination e has it at operating point 3. It is important to highlight that the assistance modulation block does not change the velocity value when the ANN outputs present one of the following errors (highlighted in bold in Table 2):

- Case a: All the outputs of the classifier indicate that there is not membership to the levels.
- Case k: Entries 1 and 3 show transition to extreme levels of muscle condition.
- Case l: Input 1 indicates level transition, input 2 indicates non-belonging, and 3 indicates belonging. With this sequence, there is no operating logic, since if input 1 shows a level change, the level that would follow is the second, but not the third.
- Case n: All inputs indicate a transition and it is not possible to know the direction of the transition.
- Cases u and x: Entries 1 and 3 show belonging to extreme levels of muscle condition.
- Case aa: All outputs of the classifier indicate that there is a membership to the levels.

**Figure 4:**
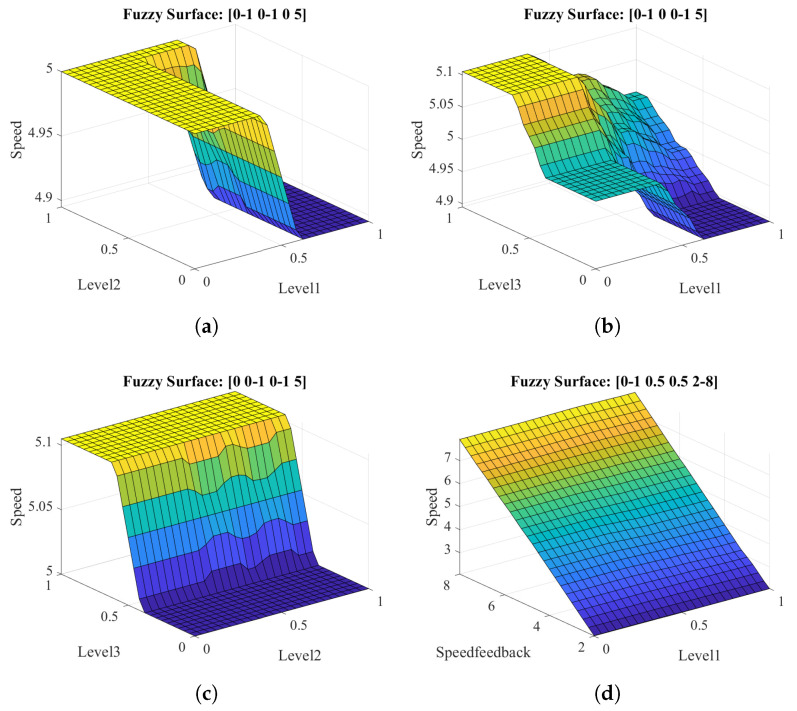
Fuzzy inference model output surface for different input combinations. (**a**) Inputs for levels 1 and 2. (**b**) Inputs for levels 1 and 3. (**c**) Inputs for levels 2 and 3. (**d**) Inputs for level 1 and velocity feedback. Each plot presents the input values in the following order: $lvl_{1}$, $lvl_{2}$, $lvl_{3}$, and ${\dot{x}}_{ref}\left(n-1\right)$.

Figure 4 presents the output surface of the assistance modulation block for some input conditions. Figure 4a–c present the fuzzy output value for changes of two inputs while the third one has a constant value of 0 and the velocity has a constant value of 5. However, the output surface presented in Figure 4d corresponds to the variation of the velocity input and one of the ANN classifiers. As shown, there is a linear relationship between input velocity and any muscular level.

A Sugeno fuzzy inference system (SFI) was implemented due to its high computational efficiency and output surface continuity, and its ability to work properly with adaptive techniques. The SFI system uses singleton output membership functions that are either constant or a linear function of the input values. Each rule in a Sugeno system calculates an output level ($z_{i}$) and a rule firing strength ($w_{i}$). Equation (3) describes the mathematical model to calculate both $z_{i}$ and $w_{i}$. Here, $x_{j}$ are the input values, *j* is the *j*th input and *i* represents the *i*th rule, while $a_{i,j}$ and $c_{i}$ are constant coefficients. The term $F{\left(\ldots \right)}_{k}$ is the membership function and *k* is the membership function used by the rule. The final output of the system is the weighted average over all the rule outputs, as described by Equation (4).

(3) $$\begin{array}{c} z_{i}=\sum_{j}^{N}\left(a_{i,j}x_{j}\right)+c_{i}\\ w_{i}={⋂}_{j=1}^{N}F{\left(j\right)}_{k} \end{array}$$

(4) $$\dot{x}=\frac{\sum_{i+1}w_{i}z_{i}}{\sum_{i+1}wi}$$

## 3. Results and Discussion

The experimental protocol was used to evaluate the performance of the integrated system: the exoskeleton and the assistance control system. The details of the implementation and evaluation stages are presented in the following sections.

### 3.1. Hardware and Control System Implementation

The exoskeleton consists of a mechanical structure of 5 pieces with 1 actuated DOF. The linear actuator L12-Actuonix is controlled by a PWM signal, where the duty cycle sets the position displacement. The development board used for the implementation was an Arduino Mega, which connects the control architecture with the exoskeleton, measures the actuator position, and generates the PWM signals. The assistance control system was implemented on a Simulink-MATLAB platform. The communication between Simulink and Arduino was done through the serial port, where Simulink sends the new duty cycle value and receives the actuator position.

Four exoskeleton fingers were constructed, to be controlled by 4 PWM signals, using 2 counters of the Arduino Mega of 16 bits with the phase and frequency correction method. Therefore, 4 active-tracking modules were implemented in Simulink with the same velocity reference, given by 1 assistance module. The myoelectric activity of the muscle and kinematic hand motion were acquired by the Myo Armband device and Leap Motion device, respectively. The Myo has a sampling frequency of 200 Hz, while the assistance control system has a sampling frequency of 1 kHz, where new data are sent at 50 Hz and received at 100 Hz.

### 3.2. Experimental Protocol

In order to design and test the architecture proposed, 2 datasets were constructed containing information regarding myoelectric activity of the muscle and kinematic hand motion. The first dataset contains signals from 4 subjects (all males, ages between 21 and 40 yr) that presented different levels of upper-limb impairment due to a stroke event [26]. The volunteers were recruited from the Brandenburgklinik Berlin-Brandenburg Hospital in Wandlitz, Germany. The experimental protocol was approved by the Ethical Committee of the Hospital, and all the participants signed the corresponding informed consent. Four types of therapies were implemented and 8 trials were acquired for each therapy: (i) open-close the hand, (ii) flexion-extension of the wrist, (iii) spread the fingers, and (iv) pinch-grip each finger. The second dataset contains information for 20 healthy subjects who reported no previous stroke events or hand impairments (all females, mean age 20 yr) [27,28]. During the experiment, 3 levels of muscular condition per subject were acquired. The experimental protocol was approved by the Research and Ethics Committee of the Engineering Faculty at Pontificia Universidad Javeriana and they accepted the corresponding informed consent. The hand movements performed by the volunteers were open-close the hand and individual finger flexion-extension. Three additional subjects with no previous stroke event or hand impairment (mean age 67 yr) were selected to evaluate the performance of the whole system.

The EMG signals were used to train and validate both the hand motion and the muscular levels. The former implemented k-nearest neighbors classifiers with a feature vector size of 136, where the accuracy using information from stroke patients was 94% for open-close the hand, 86% for flexion-extension, 92% for spread fingers, and 86% for pinch-grip [26]. The latter calculated the degree of membership to the muscular levels of the EMG signals through ANN models. As mentioned in the previous section, 3 ANN models were trained (one per muscular level) where the data for all hand movements of the second dataset were grouped. The average accuracy of these models during the testing step was 90%. The motion kinematics of the hand were used in the training module to calculate the velocity reference and angular position. This process was done for each subject individually to initialize the system with the current state of the subject.

For the experimental tests, we used the first dataset and the additional 3 new subjects from the second dataset. This allowed us to evaluate the system in two different scenarios: the first one with signals from patients with motor disabilities, and the second one with controlled changes in muscle condition. The first step was the setup of the control system through the training module, where the velocity and the angular position were calculated for different trials. Then, the real-time test was applied for each subject.

### 3.3. System Performance

In the present study, we have designed a robotic system for rehabilitation of the hand movement to be used for post-stroke patients. The experimental protocol evaluates the performance of the assistance control on the exoskeleton. Specifically, the performance corresponds to the L2 norm of the relative error of the end-effector position, the joint positions, and the actuator displacement. Furthermore, we evaluated the changes in the EMG signal to guarantee a smooth transition of the movements through position value steps between consecutive samples. The results presented here compare the performance of the model simulation and real-time experiment.

**Figure 5:**
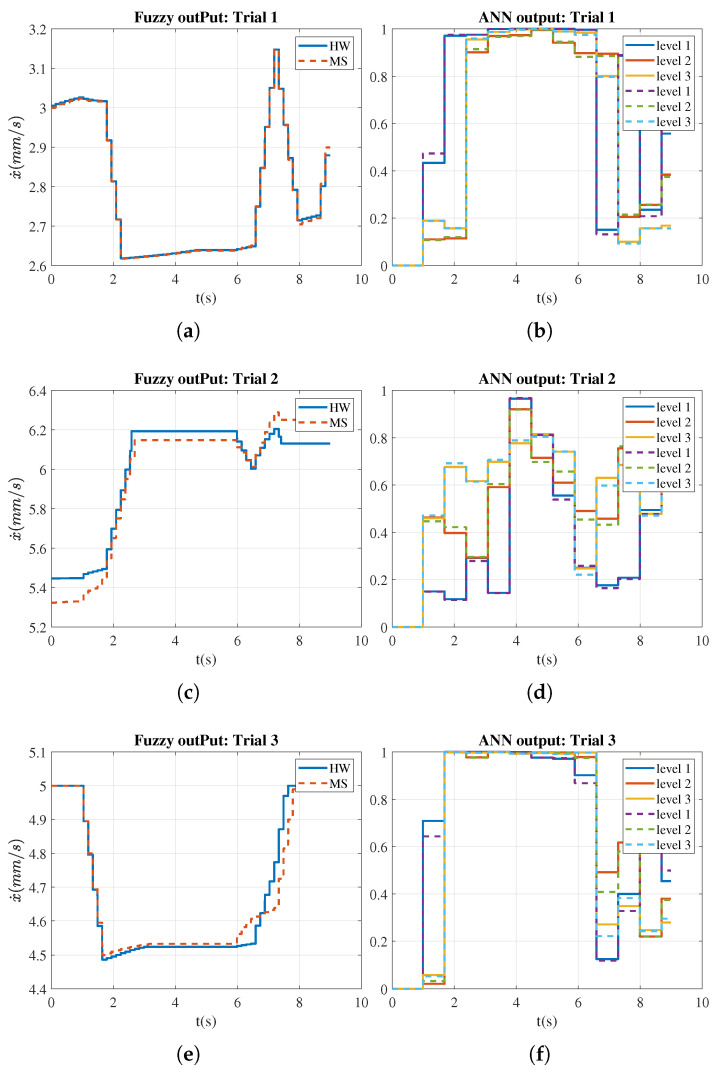
Velocity modulation (**a**,**c**,**e**) and membership degree to the 3 muscular levels (**b**,**d**,**f**). Simulation (MS, continuous line) and experimental (HW, dashed lines) were compared for each trial.

Figure 5 presents the ANN and fuzzy results for one subject. The error between simulation and experiments was approximately 0.03. This difference is mainly due to the change in the membership degree provided by the ANN classifiers and the on-off control, since the fuzzy system does not update the speed value when the hand movement is not enabled.

**Figure 6:**
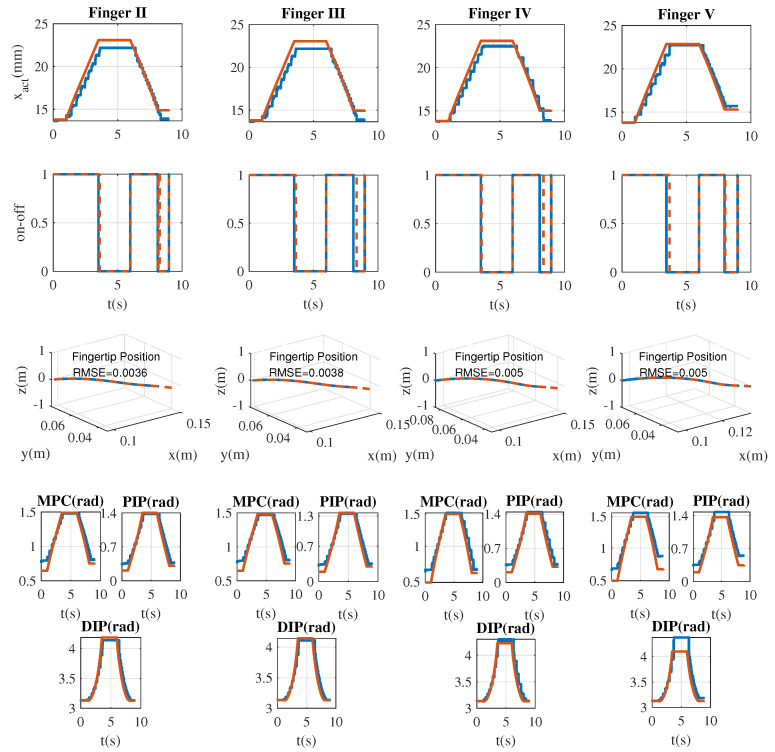
Low-level on-off position controller to drive the linear actuators of the exoskeleton for simulation (red lines) and experiments (blue lines). The first row corresponds to the actuator displacement $x_{act}$, the second row to the on-off control output, the third row to the Cartesian position of the fingertip, and the last two rows to the joint positions (MPC, PIP, and DIP).

During the tests reported in Figure 6, it was observed that the on-off controller had a higher activation during the experiments than with the simulation model. The additional activation was expected since the system depends on the real motor position feedback (moving average filter) and the serial communication, which presented delays. These variations cause the on-off control to turn on and off since the tracking error varies. The discontinuity of the actuator and the joint angular position were obtained by selecting the maximum value of the angular position difference between samples. The actuator has a step resolution of approximately 0.9 mm, with a mean value of 1 mm and a variance of 0.029 mm${}^{2}$. The angular discontinuity analysis (Table 3) shows that the DIP joint obtains the highest value with an average of 0.21 rad or 5.72${}^{\circ }$, while the MPC joint obtains the lowest value with an average of 0.04 rad or 2.29${}^{\circ }$.

In the case of the percentage error of the joint angles of the exoskeleton system, the PIP joint obtained a more significant tracking error with a mean of 18% for finger II, 17% for finger III, 19% for finger IV, and 20% for finger V. However, the DIP joint had a small tracking error with a mean of 5.6% for finger II, 3.9% for finger III, 4.4% for finger IV, and 3.9% for finger V. The experimental protocol showed that the fuzzy system could set the velocity, maintaining the output value in the range speed assigned to each muscular level. The results show that the velocity value corresponds to the fuzzy rules expected, even if the ANN outputs present an error. In addition, the system effectively modulates the movement of the exoskeleton based on myoelectric signals. In cases where the reference speed did not meet the assistance requirements, the scheme managed to bring it to the appropriate range without presenting an abrupt change in the speed profile. However, the system with the actuator feedback presented a low tracking error in the actuator displacement of around 5%. Therefore, a natural movement is guaranteed by the assistance system. The on-off controller accomplished the task of activating and deactivating the hand’s movement according to the error between the Cartesian position of the fingertip measurement and the Cartesian position reference.

## 4. Conclusions

This paper was focused on the design and implementation of an assist-as-needed robotic exoskeleton for hand rehabilitation. Muscular condition levels were used as a measurement for effort, while a closed-loop control scheme was developed to properly counterbalance muscle effort based on the characterized EMG data.

The experimental results let us conclude that the proposed control architecture solved two major challenges: (i) the adjustment of the exoskeleton-driven velocity based on the detection of muscular effort, and (ii) precise tracking and smooth response of the control output when subjected to muscular-level transitions. An ANN-fuzzy model is used to feedback the muscular condition level from the EMG readings in real-time, while a low-level position controller drives the exoskeleton accordingly. As observed from Figure 5, the fuzzy rules determined the specific velocities according to the range assigned to each muscular level. In addition, the switching among ranges was smooth and precise, yielding a maximum velocity variation of 0.7 mm/s.

In terms of tracking, the experiments reported a follow-up error in the actuator displacement around 5%, being lower than the error from the mechanical range of motion. Errors were constantly measured by comparing the Cartesian position of the fingertip against the position reference. For the calculations of the joint positions, an inverse kinematics model was used, where a negligible propagation error was achieved. Overall, the output control signal was accurately enabled when required (assist-as-needed).

Although we used several EMG signals acquired from stroke patients with hand impairments, future work will be oriented towards real clinical rehabilitation scenarios. We still have major challenges in pre-processing EMG signals directly from the electrodes in real-time. With the advent of system-on-chip (SoC) technology with neural engines capable of training and re-configuring machine learning models in real-time, a force feedback control should be derived to directly compensate muscle fatigue with force assistance. 

## Figures and Tables

**Table 1:** Modified Denavit–Hartenberg parameters for each joint. The values for $l_{P}$, $l_{M}$, and $l_{D}$ are the length of the proximal, medial, and distal. The $l_{Mp}$ is 10 cm corresponding to the actuator length.

Join	Frame	α	*a*	*q*	*d*
CMC flexion-extension	1	π/2	0	$q_{1}$	0
CMC abduction-adduction	2	0	$l_{Mp}$	$q_{2}$	0
MPC abduction-adduction	3	−π/2	0	$q_{3}$	0
MPC flexion-extension	4	0	$l_{P}$	$q_{4}$	0
PIP flexion-extension	5	0	$l_{M}$	$q_{5}$	0
DIP flexion-extension	6	0	$l_{D}$	$q_{6}$	0

**Table 2:** Combination of the inputs (ANN classifier outputs) for three states: not belonging to the corresponding muscular condition level ($N_{lvl}$), transition between muscular levels ($T_{lvl}$), and belonging to the corresponding muscular condition level ($B_{lvl}$).

Case	In1	In2	In3	Case	In1	In2	In3	Case	In1	In2	In3
**a**	$N_{lvl}$	$N_{lvl}$	$N_{lvl}$	j	$T_{lvl}$	$N_{lvl}$	$N_{lvl}$	s	$B_{lvl}$	$N_{lvl}$	$N_{lvl}$
b	$N_{lvl}$	$N_{lvl}$	$T_{lvl}$	**k**	$T_{lvl}$	$N_{lvl}$	$T_{lvl}$	t	$B_{lvl}$	$N_{lvl}$	$T_{lvl}$
c	$N_{lvl}$	$N_{lvl}$	$B_{lvl}$	**l**	$T_{lvl}$	$N_{lvl}$	$B_{lvl}$	**u**	$B_{lvl}$	$N_{lvl}$	$B_{lvl}$
d	$N_{lvl}$	$T_{lvl}$	$N_{lvl}$	m	$T_{lvl}$	$T_{lvl}$	$N_{lvl}$	v	$B_{lvl}$	$T_{lvl}$	$N_{lvl}$
e	$N_{lvl}$	$T_{lvl}$	$T_{lvl}$	**n**	$T_{lvl}$	$T_{lvl}$	$T_{lvl}$	w	$B_{lvl}$	$T_{lvl}$	$T_{lvl}$
f	$N_{lvl}$	$T_{lvl}$	$B_{lvl}$	o	$T_{lvl}$	$T_{lvl}$	$B_{lvl}$	**x**	$B_{lvl}$	$T_{lvl}$	$B_{lvl}$
g	$N_{lvl}$	$B_{lvl}$	$N_{lvl}$	p	$T_{lvl}$	$B_{lvl}$	$N_{lvl}$	y	$B_{lvl}$	$B_{lvl}$	$N_{lvl}$
h	$N_{lvl}$	$B_{lvl}$	$T_{lvl}$	q	$T_{lvl}$	$B_{lvl}$	$T_{lvl}$	z	$B_{lvl}$	$B_{lvl}$	$T_{lvl}$
i	$N_{lvl}$	$B_{lvl}$	$B_{lvl}$	r	$T_{lvl}$	$B_{lvl}$	$B_{lvl}$	**aa**	$B_{lvl}$	$B_{lvl}$	$B_{lvl}$

**Table 3:** Discontinuity of the joint angular position for fingers II–V, calculated for the 10 patients in the first dataset and the 3 additional subjects with 3 muscular levels.

Level	Subject		II			III			IV			V	
		MCP	PIP	DIP	MCP	PIP	DIP	MCP	PIP	DIP	MCP	PIP	DIP
1	1	0.03	0.055	0.099	0.028	0.051	0.109	0.03	0.043	0.099	0.031	0.062	0.123
	2	0.032	0.044	0.055	0.032	0.051	0.083	0.033	0.046	0.078	0.032	0.052	0.088
	3	0.096	0.146	0.237	0.092	0.08	0.086	0.034	0.054	0.075	0.032	0.052	0.088
2	1	0.031	0.042	0.089	0.034	0.054	0.098	0.03	0.06	0.108	0.03	0.058	0.126
	2	0.035	0.057	0.121	0.039	0.06	0.089	0.031	0.042	0.082	0.032	0.053	0.088
	3	0.031	0.058	0.094	0.037	0.055	0.082	0.031	0.05	0.069	0.033	0.063	0.101
3	1	0.03	0.044	0.104	0.04	0.057	0.095	0.036	0.065	0.093	0.038	0.076	0.133
	2	0.037	0.053	0.084	0.032	0.058	0.13	0.031	0.046	0.082	0.032	0.057	0.088
	3	0.035	0.06	0.085	0.031	0.061	0.114	0.033	0.051	0.106	0.031	0.053	0.088
Patients	1	0.033	0.056	0.081	0.032	0.058	0.1	0.032	0.045	0.073	0.028	0.054	0.094
	2	0.033	0.068	0.14	0.031	0.058	0.097	0.029	0.045	0.088	0.031	0.056	0.11
	3	0.064	0.067	0.106	0.033	0.06	0.088	0.029	0.041	0.075	0.03	0.06	0.114
	4	0.033	0.057	0.091	0.034	0.066	0.123	0.031	0.049	0.068	0.036	0.066	0.104
	5	0.03	0.049	0.087	0.035	0.053	0.089	0.027	0.039	0.076	0.028	0.056	0.104
	6	0.03	0.048	0.073	0.033	0.056	0.08	0.03	0.039	0.053	0.036	0.072	0.132
	7	0.032	0.047	0.076	0.034	0.048	0.081	0.031	0.036	0.047	0.032	0.046	0.094
	8	0.048	0.053	0.071	0.033	0.058	0.093	0.031	0.039	0.059	0.032	0.057	0.115
	9	0.035	0.049	0.053	0.032	0.05	0.1	0.031	0.04	0.059	0.032	0.057	0.115
	10	0.033	0.06	0.093	0.037	0.052	0.081	0.03	0.044	0.059	0.032	0.057	0.114

## Appendix A

Table A1 presents the output velocity of the assistance modulation according to the level combinations from Table 2 and the velocity input.

**Table A1:** Velocity regulation according to the input combination from Table 2 and the previous velocity value (${\dot{x}}_{ref}\left(n-1\right)$). The final decision could be to maintain speed ($S_{p}$), increase speed ($S_{p}+\Delta_{S}$), or decrease speed ($S_{p}-\Delta_{S}$).

Case	$S_{p}={\dot{x}}_{\mathrm{input}}$												
	$S_{1}$	$\left(S_{1},S_{2}\right)$	$S_{2}$	$\left(S_{2},S_{3}\right)$	$S_{3}$	$\left(S_{3},S_{4}\right)$	$S_{4}$	$\left(S_{4},S_{5}\right)$	$S_{5}$	$\left(S_{5},S_{6}\right)$	$S_{6}$	$\left(S_{6},S_{7}\right)$	$S_{7}$
a	$S_{p}$		$S_{p}$		$S_{p}$		$S_{p}$		$S_{p}$		$S_{p}$		$S_{p}$
b	$S_{p}+\Delta_{S}$		$S_{p}$	$S_{p}+\Delta_{S}$	$S_{p}-\Delta_{S}$		$S_{p}-\Delta_{S}$		$S_{p}-\Delta_{S}$		$S_{p}-\Delta_{S}$		$S_{p}-\Delta_{S}$
c	$S_{p}$	$S_{p}-\Delta_{S}$	$S_{p}-\Delta_{S}$		$S_{p}-\Delta_{S}$		$S_{p}-\Delta_{S}$		$S_{p}-\Delta_{S}$		$S_{p}-\Delta_{S}$		$S_{p}-\Delta_{S}$
d	$S_{p}+\Delta_{S}$		$S_{p}+\Delta_{S}$		$S_{p}+\Delta_{S}$		$S_{p}$	$S_{p}-\Delta_{S}$	$S_{p}-\Delta_{S}$		$S_{p}-\Delta_{S}$		$S_{p}-\Delta_{S}$
e	$S_{p}+\Delta_{S}$		$S_{p}+\Delta_{S}$		$S_{p}$	$S_{p}-\Delta_{S}$	$S_{p}-\Delta_{S}$		$S_{p}-\Delta_{S}$		$S_{p}-\Delta_{S}$		$S_{p}-\Delta_{S}$
f	$S_{p}+\Delta_{S}$		$S_{p}$	$S_{p}-\Delta_{S}$	$S_{p}-\Delta_{S}$		$S_{p}-\Delta_{S}$		$S_{p}-\Delta_{S}$		$S_{p}-\Delta_{S}$		$S_{p}-\Delta_{S}$
g	$S_{p}+\Delta_{S}$		$S_{p}+\Delta_{S}$		$S_{p}+\Delta_{S}$		$S_{p}$	$S_{p}-\Delta_{S}$	$S_{p}-\Delta_{S}$		$S_{p}-\Delta_{S}$		$S_{p}-\Delta_{S}$
h	$S_{p}+\Delta_{S}$		$S_{p}+\Delta_{S}$		$S_{p}+\Delta_{S}$		$S_{p}$	$S_{p}-\Delta_{S}$	$S_{p}-\Delta_{S}$		$S_{p}-\Delta_{S}$		$S_{p}-\Delta_{S}$
i	$S_{p}+\Delta_{S}$		$S_{p}+\Delta_{S}$		$S_{p}$	$S_{p}+\Delta_{S}$	$S_{p}-\Delta_{S}$		$S_{p}-\Delta_{S}$		$S_{p}-\Delta_{S}$		$S_{p}-\Delta_{S}$
j	$S_{p}+\Delta_{S}$		$S_{p}+\Delta_{S}$		$S_{p}+\Delta_{S}$		$S_{p}+\Delta_{S}$		$S_{p}+\Delta_{S}$		$S_{p}$	$S_{p}-\Delta_{S}$	$S_{p}-\Delta_{S}$
k	$S_{p}$		$S_{p}$		$S_{p}$		$S_{p}$		$S_{p}$		$S_{p}$		$S_{p}$
l	$S_{p}+\Delta_{S}$		$S_{p}$	$S_{p}-\Delta_{S}$	$S_{p}-\Delta_{S}$		$S_{p}-\Delta_{S}$		$S_{p}-\Delta_{S}$		$S_{p}-\Delta_{S}$		$S_{p}-\Delta_{S}$
m	$S_{p}+\Delta_{S}$		$S_{p}+\Delta_{S}$		$S_{p}+\Delta_{S}$		$S_{p}+\Delta_{S}$		$S_{p}$	$S_{p}-\Delta_{S}$	$S_{p}-\Delta_{S}$		$S_{p}-\Delta_{S}$
n	$S_{p}$		$S_{p}$		$S_{p}$		$S_{p}$		$S_{p}$		$S_{p}$		$S_{p}$
o	$S_{p}$		$S_{p}$		$S_{p}$		$S_{p}$		$S_{p}-\Delta_{S}$		$S_{p}-\Delta_{S}$		$S_{p}-\Delta_{S}$
p	$S_{p}+\Delta_{S}$		$S_{p}+\Delta_{S}$		$S_{p}+\Delta_{S}$		$S_{p}+\Delta_{S}$		$S_{p}+\Delta_{S}$		$S_{p}$	$S_{p}-\Delta_{S}$	$S_{p}-\Delta_{S}$
q	$S_{p}+\Delta_{S}$		$S_{p}+\Delta_{S}$		$S_{p}+\Delta_{S}$		$S_{p}$	$S_{p}-\Delta_{S}$	$S_{p}-\Delta_{S}$		$S_{p}-\Delta_{S}$		$S_{p}-\Delta_{S}$
r	$S_{p}+\Delta_{S}$		$S_{p}+\Delta_{S}$		$S_{p}$	$S_{p}-\Delta_{S}$	$S_{p}-\Delta_{S}$		$S_{p}-\Delta_{S}$		$S_{p}-\Delta_{S}$		$S_{p}-\Delta_{S}$
s	$S_{p}+\Delta_{S}$		$S_{p}+\Delta_{S}$		$S_{p}+\Delta_{S}$		$S_{p}+\Delta_{S}$		$S_{p}+\Delta_{S}$		$S_{p}+\Delta_{S}$		$S_{p}$
t	$S_{p}+\Delta_{S}$		$S_{p}+\Delta_{S}$		$S_{p}+\Delta_{S}$		$S_{p}+\Delta_{S}$		$S_{p}+\Delta_{S}$		$S_{p}$	$S_{p}-\Delta_{S}$	$S_{p}-\Delta_{S}$
u	$S_{p}$		$S_{p}$		$S_{p}$		$S_{p}$		$S_{p}$		$S_{p}$		$S_{p}$
v	$S_{p}+\Delta_{S}$		$S_{p}+\Delta_{S}$		$S_{p}+\Delta_{S}$		$S_{p}+\Delta_{S}$		$S_{p}+\Delta_{S}$		$S_{p}$	$S_{p}-\Delta_{S}$	$S_{p}-\Delta_{S}$
w	$S_{p}+\Delta_{S}$		$S_{p}+\Delta_{S}$		$S_{p}+\Delta_{S}$		$S_{p}$		$S_{p}-\Delta_{S}$		$S_{p}-\Delta_{S}$		$S_{p}-\Delta_{S}$
x	$S_{p}$		$S_{p}$		$S_{p}$		$S_{p}$		$S_{p}$		$S_{p}$		$S_{p}$
y	$S_{p}+\Delta_{S}$		$S_{p}+\Delta_{S}$		$S_{p}+\Delta_{S}$		$S_{p}+\Delta_{S}$		$S_{p}$	$S_{p}-\Delta_{S}$	$S_{p}-\Delta_{S}$		$S_{p}-\Delta_{S}$
z	$S_{p}+\Delta_{S}$		$S_{p}+\Delta_{S}$		$S_{p}+\Delta_{S}$		$S_{p}+\Delta_{S}$		$S_{p}$	$S_{p}-\Delta_{S}$	$S_{p}-\Delta_{S}$		$S_{p}-\Delta_{S}$
aa	$S_{p}$		$S_{p}$		$S_{p}$		$S_{p}$		$S_{p}$		$S_{p}$		$S_{p}$
